# Measuring Similarity among Protein Sequences Using a New Descriptor

**DOI:** 10.1155/2019/2796971

**Published:** 2019-11-22

**Authors:** Mervat M. Abo-Elkhier, Marwa A. Abd Elwahaab, Moheb I. Abo El Maaty

**Affiliations:** Department of Engineering Mathematics and Physics, Faculty of Engineering, Mansoura University, Mansoura 35516, Egypt

## Abstract

The comparison of protein sequences according to similarity is a fundamental aspect of today's biomedical research. With the developments of sequencing technologies, a large number of protein sequences increase exponentially in the public databases. Famous sequences' comparison methods are alignment based. They generally give excellent results when the sequences under study are closely related and they are time consuming. Herein, a new alignment-free method is introduced. Our technique depends on a new graphical representation and descriptor. The graphical representation of protein sequence is a simple way to visualize protein sequences. The descriptor compresses the primary sequence into a single vector composed of only two values. Our approach gives good results with both short and long sequences within a little computation time. It is applied on nine beta globin, nine ND5 (NADH dehydrogenase subunit 5), and 24 spike protein sequences. Correlation and significance analyses are also introduced to compare our similarity/dissimilarity results with others' approaches, results, and sequence homology.

## 1. Introduction

Information encoded in the genome of any organism plays a central role in defining the life of that organism. The nucleotide sequence that forms any gene is translated into its corresponding amino acid sequence. This sequence of amino acids becomes functional only when it adopts its tertiary structure. Experimental methods such as X-ray diffraction and nuclear magnetic resonance are considered authoritative ways for obtaining proteins' structure and function. These experimental methods are very expensive and time consuming. Therefore, computational methods for predicting protein structure have become very useful. Proteins with similar sequences are usually homologous, typically displaying similar 3D structure and function.

Sequence alignment is the first step of 3D structure prediction for protein sequences. Alignment approaches are classified into alignment-based and alignment-free methods. BLAST (basic local alignment search tool) and ClustalW are the most widely used computer programs for alignment-based approaches [1–3]. Results of these programs provide an approximate solution to the protein alignment problem. On the other hand, many alignment-free approaches are proposed for sequence comparison. Most biological sequence analysis methods still have weaknesses, including having low precision and being time consuming [4, 5].

Similarity/dissimilarity analysis of biological sequences is used to extract information stored in the protein sequence. Many mathematical schemes have been proposed to this end. Graphical representations of biological sequences identify the information content of any sequence to help biologists choose another complex theoretical or experimental method. Graphical representation provides not only visual qualitative inspection of gene data but also mathematical characterizations through objects such as matrices.

Some 2D and 3D graphical representations are created by selecting a geometrical object that is used to describe nucleic acid bases or residues [6–10]. Others are based on assigning vectors of two or three components to nucleic acid bases or amino acids [11–17]. Adjacency matrices are also introduced in some articles [18–21], where an exact solution is obtained to the protein alignment problem. Additional methods use discrete Fourier transform (DFT) in which DNA sequences are mapped into four binary indicator sequences, followed by the application of DFT on these indicator sequences to transform them into a frequency domain [22, 23]. Dynamic representation is used to remove degeneracies in the previously mentioned approaches [24–31]. Another method is based on the simplified pulse-coupled neural network (S-PCNN) and Huffman coding where the triplet code was used as a code bit to transform DNA sequence into numerical sequence [32].

In this study, we introduce a new alignment-free method for protein sequences. Each amino acid in the protein sequence is represented by a number, and a new 2D graphical representation is suggested. A new descriptor is introduced, comprising a vector composed of the mean and standard deviation of the total numbers of each protein sequence (${\bar{A}}_{t}$, SA_*t*_). Our graphical representation eliminates degeneracy and has no loss of information. It is suitable for both short and long sequences. As a proof of concept, our approach is applied on nine beta globin protein sequences and nine ND5 (NADH dehydrogenase subunit 5) protein sequences. It can be applied on any sequence length with the same efficiency. Correlation and significance analyses are introduced among our results, along with PID% [15] and ClustalW [33] to demonstrate the utility of our approach.

## 2. Dataset, Technology, and Tools

All the protein sequences used in this study were downloaded from The National Center for Biotechnology Information (NCBI) “https://www.ncbi.nlm.nih.gov” as FASTA files. These FASTA files are imported into Wolfram Mathematica 8 where all the results and figures are produced. They are nine beta globin, nine ND5 (NADH dehydrogenase subunit 5), and 24 coronaviruses protein sequences as illustrated in Tables 1–3, respectively. These datasets are selected to be different in length.

## 3. 2D Graphical Representation

A new 2D graphical representation is introduced. Each amino acid in any protein sequence is represented by the suggested intensity  *Y*_*x* _(*i*) and intensity level  *A*_*x*  _(*i*). The intensity (*Y*_*x* _(*i*)) of each amino in the sequence depends on its abundance and location in the different sequences. It is calculated using

(1) $$\begin{array}{c} Y_{x\;}\left(i\right)=f_{x}i, \end{array}$$

where *f*_*x*_ is the frequency of amino acid *x* in the sequence, number of times of *x*/*N*. *N* is the protein sequence length, number of residues in protein sequence. *i* is the position of each amino acid *x* in a sequence.

Then, the intensity level *A*_*x*_(*i*) of each amino acid (*x*) in the sequence is calculated by using the natural logarithm function as in

(2) $$\begin{array}{c} A_{x}\left(i\right)=\left\{\begin{array}{cc} \left|10\;\ln\left(\frac{\;Y_{x}\left(i\right)}{N}\right)\right|, & \text{if the element }x\text{ exists at position }i,\\ 0, & \text{if the element }x\text{ does not exist at position }i. \end{array}\right. \end{array}$$

Therefore, each amino acid has its own intensity level which is a vector of *N* elements according to equation (2). Finally, the combined intensity level of the protein sequence *A*_*t*_(*i*) is obtained by the summation of the 20 intensity levels' vectors *A*_*x*_(*i*) of the protein sequence by using equation (3). The combined intensity level *A*_*t*_(*i*) is also a vector of *N* elements:

(3) $$\begin{array}{c} A_{t}\left(i\right)=\overset{20}{\underset{x=1}{\sum }}A_{x}\left(i\right). \end{array}$$

Each amino acid has its own graph. Now, twenty graphs are obtained for each sequence of the 20 different amino acids. The combined graph is obtained by combining these 20 graphs within a single graph. This combined intensity level is our new 2D graphical representation.

Our approach is first applied on two short segments of protein from “yeast *Saccharomyces cerevisiae*”:

  Protein I: “WTFESRNDPAKDPVILWLNGGPGCS‐SLTGL”
  Protein II: “WFFESRNDPANDPIILWLNGGPGCS‐SFTGL”

These two short proteins consist of 30 amino acids each. The two sequences are different in amino acids at positions 2, 11, 14, and 27. The values *Y*_*x*_(*i*) and *A*_*x*_(*i*) for each amino acid in the two sequences are calculated. For protein I, the G amino acid is repeated four times in the protein sequence. These four repeats occur in positions 20, 21, 23, and 29. The frequency, *f*_G_, equals (4/30). By substituting in equations (1) and (2), the results of *Y*_*G* _(*i*) and *A*_*G*_(*i*) are presented in Table 4.

**Figure 1:**
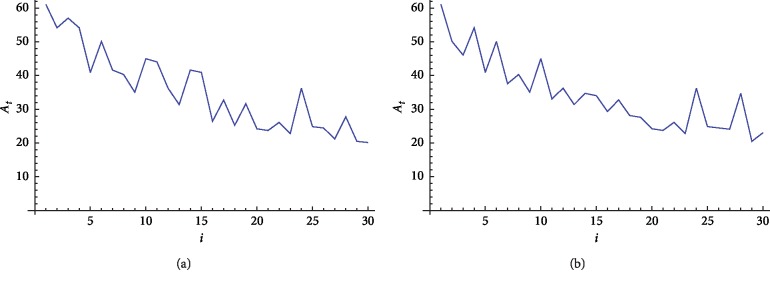
2D graphical representation of the “combined intensity level” of two short segments of protein of “yeast *Saccharomyces cerevisiae*”. (a) Protein 1. (b) Protein 2.

By summing the values of *A*_*x*_(*i*) for all amino acids in protein I, the total value of *A*_*t*_(*i*) is obtained, as shown in Figure 1(a). The position *i* of each amino acid is located on the *x*-axis, and the total intensity level *A*_*t*_(*i*) is located on the *y*-axis. Figures 1(a) and 1(b) show the intensity level of protein I and protein II, respectively. Of note, the two graphs have different *A*_*x*_(*i*) values at positions 2, 11, 14, and 27.

**Figure 2:**
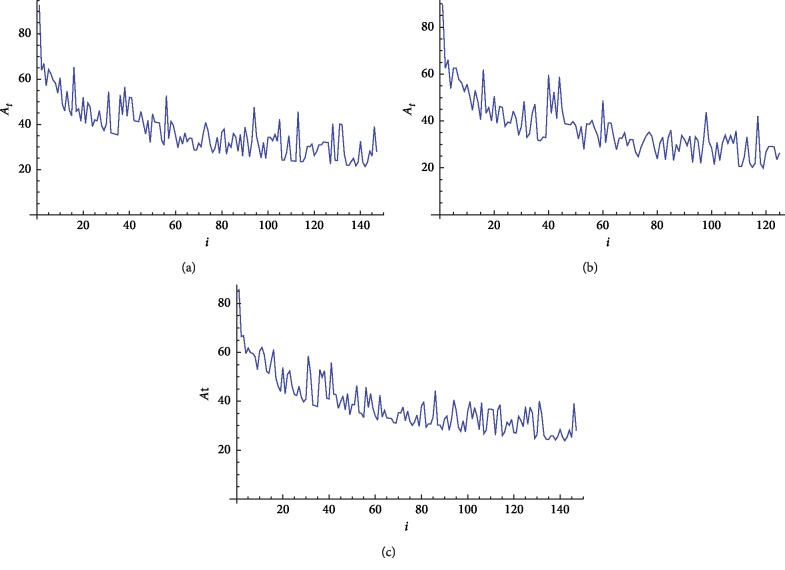
2D graphical representation the “combined intensity level” of beta globin protein sequences. (a) Human, (b) chimpanzee, and (c) opossum.

**Figure 3:**
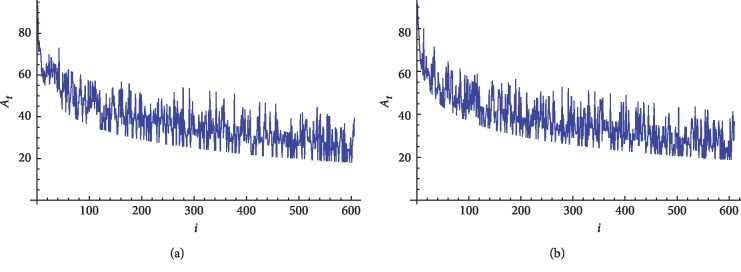
2D graphical representation of “combined intensity level” of ND5 protein sequences. (a) Fin whale and (b) rat.

We next apply our approach on nine beta globin and nine ND5 (NADH dehydrogenase subunit 5) protein sequences, which are illustrated in Tables 1 and 2. The 2D graphical representation for human, chimpanzee, and opossum beta globin protein sequences is illustrated in Figures 2(a)–2(c), respectively. The 2D graphical representations for fin whale and rat ND5 protein sequences are illustrated in Figures 3(a) and 3(b), respectively.

**Figure 4:**
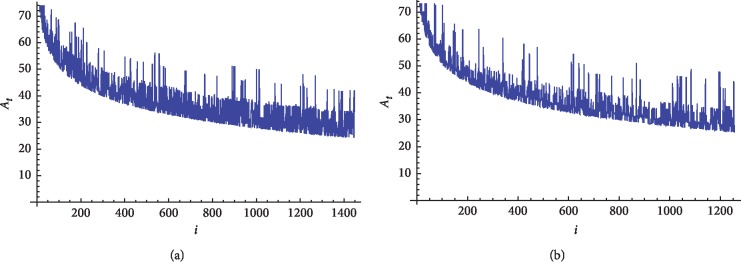
2D graphical representation of “combined intensity level” of TGEVG and GD03T0013 coronaviruses protein sequences. (a) Fin whale and (b) rat.

We finally apply our approach on 24 coronaviruses protein sequences which are illustrated in Table 3. The 2D graphical representation of TGEVG from class I and GD03T0013 from SARS_CoV protein sequences is illustrated in Figures 4(a) and 4(b) respectively.

## 4. Protein Sequence Descriptor

Mathematical descriptors help in recognizing major differences among similar protein sequences quantitatively. A new descriptor for protein sequences is suggested, which is a vector composed of the arithmetic mean ${\bar{A}}_{t}\;$ and standard deviation *SA*_*t*_ of the combined intensity level value *A*_*t*_(*i*) of the protein sequence. They are evaluated according to the following equations:

(4) $$\begin{array}{c} \bar{A_{t}}=\frac{1}{N}\overset{i=N}{\underset{i=1}{\sum }}A_{t}\left(i\right),\\ {\text{SA}}_{t}=\frac{1}{N-1}{\sqrt{\left(A_{t}\left(i\right)-\bar{A_{t}}\right)}}^{2}. \end{array}$$

This descriptor compresses the information from primary protein sequences into a single vector composed of only two values. The beta globin, ND5, and coronaviruses protein sequence descriptors are illustrated in Tables 5–7, respectively.

**Figure 5:**
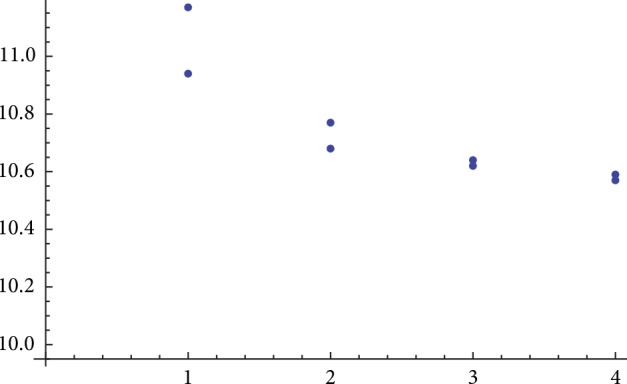
The four classes of the 24 coronaviruses protein sequences based on their standard deviation of the combined intensity level.

Table 7 shows that the mean of all 24 coronaviruses is around 38.7 and with a range from 38.601 to 38.838 while their standard deviation varies according to their class. They are divided into four classes. The first four viruses belong to class I. The fifth to the ninth coronaviruses belong to class II. Class III contains the tenth and eleventh viruses. The rest viruses from the 12th to the 24th belong to SARS-CoV. According to our approach, the standard deviation of class I ranges from 10.94 to 11.17. Class II's standard deviation ranges from 10.68 to 10.77. Class III's standard deviation has values from 10.6271 to 10.6458. SARS-CoV's standard deviation almost equals 10.58. The resulting standard deviation values of the 24 coronaviruses classify them correctly to the four classes. The coronaviruses classes' ranges according to our approach are shown in Figure 5.

## 5. Similarity/Dissimilarity Analysis

To compare the species' protein sequences, the Euclidean distance among species' descriptors is evaluated. For example, the human beta globin protein sequence's descriptor is (37.145, 11.505) and the chimpanzee beta globin protein sequence's descriptor is (36.912, 11.586). To measure the degree of similarity between human and chimpanzee, the Euclidean distance between these vectors is evaluated. The similarity/dissimilarity matrices of beta globin and ND5 protein sequences are illustrated in Tables 8 and 9, respectively.  Table 8 results show that human and chimpanzee sequences are similar. There is also striking similarity between mouse and rat sequences, while human and opossum sequences are obviously dissimilar.  Table 9 results show that pigmy chimpanzee, common chimpanzee, human, and gorilla ND5 protein sequences are similar, while the blue whale is similar to the fin whale, and mouse is similar to rat. Similar to the other sequence, human and opossum are still dissimilar. However, our algorithm cannot measure the degree of similarity very well for pigmy chimpanzee. The distance between human and pigmy chimpanzee is 0.1826, while the distance between human and gorilla is 0.0575, as shown in Table 9. The results of both Tables 8 and 9 are approximately comparable to previous reports [13, 15, 21, 33–39].

## 6. The Phylogenetic Tree of the Protein Sequences Based on Our Method

**Figure 6:**
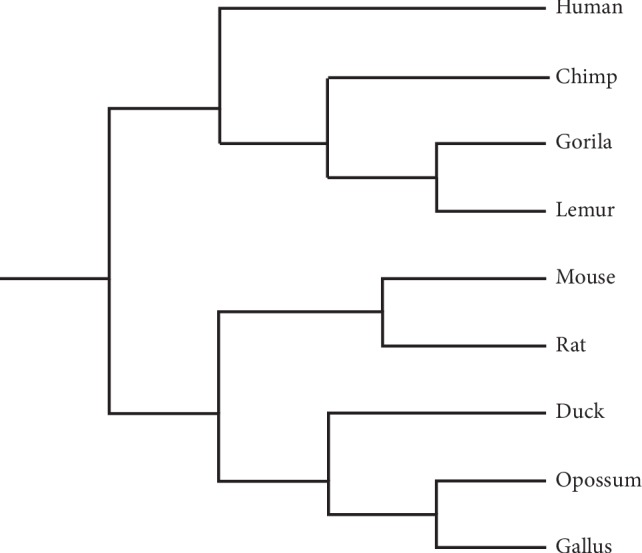
The phylogenetic tree of the nine beta globin protein sequences based on our method.

**Figure 7:**
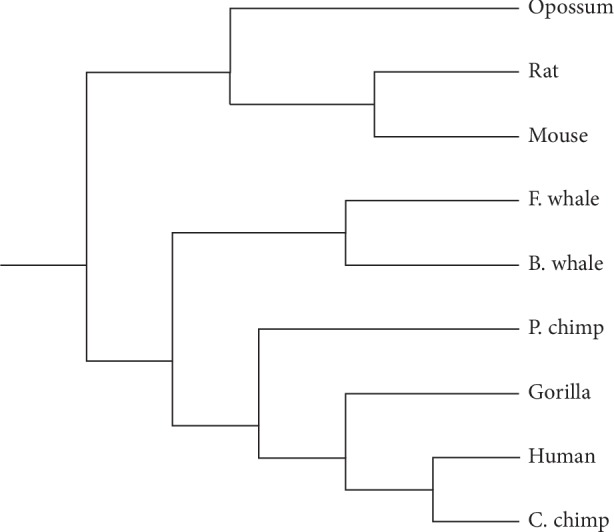
The phylogenetic tree of the nine ND5 protein sequences based on our method.

We got the phylogenetic trees of beta globin and ND5 protein sequences by applying the UPGMA (Unweighted Pair Group Method with Arithmetic Mean). The phylogenetic tree based on Tables 8 and 9 of our method is presented in Figures 6 and 7, respectively.  Figure 6 proves the utility of our similarity/dissimilarity analysis for beta globin protein sequences.  Figure 7 shows our analysis of similarity/dissimilarity of ND5. It is mentioned that our algorithm cannot measure the degree of similarity very well for pigmy chimpanzee with human. This appears of course in Figure 7. The P. chimp branch should be close to C. chimp. Despite this error, the tree shows that human, common chimpanzee, pigmy chimpanzee, and gorilla belong to the same cluster. To check the effect of this error on our algorithm, the results of our algorithm are compared to sequence homology. A correlation and significance analysis is also provided.

## 7. Our Method Compared to PID% and ClustalW Results

The results of our algorithm are compared to the sequence homology by two methods. First, we use the Smith Waterman algorithm to calculate the number of identical residues in each pair of protein sequences [15]. The results of the PID% of nine beta globin sequences are illustrated as a similarity/dissimilarity matrix in Table 10. The larger PID% represents the more similar protein sequences. A correlation and significance analysis is provided to compare our approach in Table 8 with PID% in Table 10. The correlation of the two sets of data is sufficiently strong when the correlation coefficient (*r*) is greater than 0.7. The negative sign of (*r*) indicates that when the first data set increases, the second data set decreases. We then assess statistical significance for correlation coefficient values greater than 0.7 to ensure that they likely do not occur by chance. Our sample set is composed of nine protein sequences. Therefore, we use 7 degrees of freedom. A *t*-value of 2.385 or greater indicates that a less than 0.05 chance of the results occurred by coincidence. The results for correlation coefficients and *t*-values for our approach are illustrated in Table 11.

Second, ClustalW is a widely used system for aligning any number of homologous nucleotides or protein sequences [33]. The ClustalW program's distance matrix of nine ND5 protein sequences is illustrated in Table 12. Correlation and significance analyses are also provided to compare our approach in Table 9 with ClustalW results in Table 12. The results of the correlation and significance analyses of our approach and other approaches [15, 33] are illustrated in Table 13. Our sample set of ND5 is also composed of nine protein sequences. Therefore, we use 7 degrees of freedom and a *t*-value of 2.385 or greater. Despite the unusual result for pigmy chimpanzee that appeared in Table 9, the correlation coefficient of pigmy chimpanzee in our similarity matrix and clustalW matrix is 0.8811. This value likely does not occur by chance, as the *t*-value equals 4.928, as illustrated in Table 13. The comparison between our results and both PID% and ClustalW and other approaches' results indicate the utility of our approach.

## 8. Conclusions

A new graphical representation of protein sequences is introduced. It is the combined intensity level of the 20 amino acids composing any protein sequence. Each amino acid in a given protein sequence has its own intensity and intensity level. They are vectors of N elements as N is the protein sequence length. The combined intensity level is then computed and graphed to represent any protein sequence graphically. Our 2D graphical representation effectively displays differences between protein sequences without degeneracies. The graph does not overlap or intersect with itself. Our new descriptor suggested a vector of two elements, which are the mean and standard deviation of the combined intensity level (${\bar{A}}_{t}\;$ and SA_*t*_). A similarity/dissimilarity analysis is evaluated by computing Euclidean distance between each two species' descriptors. Examination of similarity/dissimilarity among nine beta globin, nine ND5, and 24 coronaviruses protein sequences provided good results compared to previous approaches. The suggested approach is effective for both short and long sequences, and the computations are very simple. Furthermore, loss of sequence information is avoided. Correlation and significance analyses with PID% and ClustalW are also introduced to show the utility of our approach.

## Figures and Tables

**Table 1:** Nine beta globin protein sequences.

No.	Species	ID	Length
1	Gorilla	CAA43421	121
2	Chimp	CAA26204	125
3	Human	AAA16334	147
4	Rat	CAA29887	147
5	Mouse	CAA24101	147
6	Gutta	ACH46399	147
7	Duck	CAA33756	147
8	Gallus	CAA23700	147
9	Opossum	AAA30976	147

**Table 2:** Nine ND5 protein sequences.

No.	Species	ID	Length
1	Human	AP_000649	603
2	Gorilla	NP_008222	603
3	Common chimpanzee	NP_008196	603
4	Pygmy chimpanzee	NP_008209	603
5	Fin whale	NP_006899	606
6	Blue whale	NP_007066	606
7	Rat	AP_004902	610
8	Mouse	NP_904338	607
9	Opossum	NP_007105	602

**Table 3:** The 24 coronaviruses protein sequences.

No.	Access no.	Abbreviation	Length	Class
1	CAB91145	TGEVG	1447	I
2	NP058424	TGEV	1447	I
3	AAK38656	PEDVC	1383	I
4	NP598310	PEDV	1383	I
5	NP937950	HCoVOC43	1361	II
6	AAK83356	BCoVE	1363	II
7	AAL57308	BCoVL	1363	II
8	AAA66399	BCoVM	1363	II
9	AAL40400	BCoVQ	1363	II
10	AAS00080	IBVC	1169	III
11	NP 040831	IBV	1162	III
12	AAS10463	GD03T0013	1255	SARS-CoV
13	AAU93318	PC4127	1255	SARS-CoV
14	AAV49720	PC4137	1255	SARS-CoV
15	AAU93319	PC4205	1255	SARS-CoV
16	AAU04646	civet007	1255	SARS-CoV
17	AAU04649	civet010	1255	SARS-CoV
18	AAV91631	A022	1255	SARS-CoV
19	AAP51227	GD01	1255	SARS-CoV
20	AAS00003	GZ02	1255	SARS-CoV
21	AAP30030	BJ01	1255	SARS-CoV
22	AAP50485	FRA	1255	SARS-CoV
23	AAP41037	TOR2	1255	SARS-CoV
24	AAQ01597	TaiwanTC1	1255	SARS-CoV

**Table 4:** The intensity and intensity level vectors of the two short segments of protein from “yeast *Saccharomyces cerevisiae*” protein sequences.

*i*	1	2	3	4	5	6	7	8	9	10	11	12	13	14	15
*Y*_*G* _(*i*)	0	0	0	0	0	0	0	0	0	0	0	0	0	0	0
*A*_*G*_(*i*)	0	0	0	0	0	0	0	0	0	0	0	0	0	0	0
*i*	16	17	18	19	20	21	22	23	24	25	26	27	28	29	30
*Y*_*G* _(*i*)	0	0	0	0	2.67	2.80	0	3.07	0	0	0	0	0	3.87	0
*A*_*G*_(*i*)	0	0	0	0	24.2	23.7	0	22.8	0	0	0	0	0	20.5	0

**Table 5:** Mean and standard deviation descriptor of beta globin protein sequences.

No.	Species	${\bar{A}}_{t}$	SA_*t*_
1	Gorilla	36.803	11.744
2	Chimp	36.912	11.586
3	Human	37.145	11.505
4	Rat	37.721	11.399
5	Mouse	37.695	11.727
6	Gutta	38.046	11.537
7	Duck	38.244	11.399
8	Gallus	38.349	11.169
9	Opossum	38.418	10.944

**Table 6:** Mean and standard deviation descriptor of ND5 protein sequences.

No.	Species	${\bar{A}}_{t}$	SA_*t*_
1	Human	37.300	12.267
2	Gorilla	37.338	12.223
3	Pigmy chimpanzee	37.249	12.091
4	Common chimpanzee	37.251	12.277
5	Fin whale	37.540	11.961
6	Blue whale	37.534	12.027
7	Rat	37.385	11.621
8	Mouse	37.328	11.562
9	Opossum	37.558	11.419

**Table 7:** Mean and standard deviation descriptor of the coronaviruses protein sequences.

	Abb.	Class no.	Mean	Standard deviation
1	TGEVG	I	38.643	10.9412
2	TGEV	I	38.643	10.9412
3	PEDVC	I	38.452	11.1723
4	PEDV	I	38.452	11.1723
5	HCoVOC43	II	38.703	10.7564
6	BCoVE	II	38.668	10.6803
7	BCoVL	II	38.678	10.6846
8	BCoVM	II	38.698	10.7755
9	BCoVQ	II	38.714	10.7656
10	IBVC	III	38.601	10.6271
11	IBV	III	38.654	10.6458
12	GD03T0013	SARS-CoV	38.833	10.5783
13	PC4127	SARS-CoV	38.838	10.5744
14	PC4137	SARS-CoV	38.832	10.5785
15	PC4205	SARS-CoV	38.838	10.5733
16	civet007	SARS-CoV	38.831	10.587
17	civet010	SARS-CoV	38.833	10.5829
18	A022	SARS-CoV	38.829	10.5892
19	GD01	SARS-CoV	38.821	10.5946
20	GZ02	SARS-CoV	38.824	10.5867
21	BJ01	SARS-CoV	38.816	10.5912
22	FRA	SARS-CoV	38.8189	10.5875
23	TOR2	SARS-CoV	38.8186	10.5932
24	TaiwanTC1	SARS-CoV	38.8176	10.5928

**Table 8:** Similarity/dissimilarity analysis among nine beta globin protein sequences.

	Human	Gorilla	Chimp	Lemur	Mouse	Rat	Opossum	Duck	Gallus
Human	0	0.417	0.246	0.461	0.593	0.586	1.391	1.104	1.25
Gorilla		0	0.192	0.104	0.892	0.980	1.802	1.481	1.649
Chimp			0	0.270	0.795	0.829	1.637	1.344	1.496
Lemur				0	0.870	0.993	1.823	1.479	1.660
Mouse					0	0.329	1.066	0.639	0.860
Rat						0	0.833	0.523	0.669
Opossum							0	0.488	0.236
Duck								0	0.253
Gallus									0

**Table 9:** Similarity/dissimilarity analysis among nine ND5 protein sequences.

	Human	Gorilla	P. chimp	C. chimp	F. whale	B. whale	Rat	Mouse	Opossum
Human	0	0.0575	0.1826	0.0503	0.3885	0.3349	0.6509	0.7054	0.8853
Gorilla		0	0.1590	0.1021	0.3311	0.2775	0.6039	0.6617	0.8332
P. chimp			0	0.1855	0.3184	0.2918	0.4890	0.5351	0.7389
C. chimp				0	0.4281	0.3776	0.6689	0.7189	0.9102
F. whale					0	0.0663	0.3737	0.4524	0.5417
B. whale						0	0.4325	0.5092	0.6079
Rat							0	0.0826	0.2656
Mouse								0	0.2705
Opossum									0

**Table 10:** The similarity distance for nine different species of beta globin proteins calculated by the PID%.

	Human	Gorilla	Chimp	Lemur	Mouse	Rat	Opossum	Duck	Gallus
Human	100	98.347	93.6	66.667	60.544	59.184	44.898	39.456	38.776
Gorilla		100	95.041	66.942	58.678	55.372	46.281	39.669	38.843
Chimp			100	61.6	55.2	52.	40.	36.8	36.
Lemur				100	53.061	48.979	40.136	31.973	31.293
Mouse					100	78.231	39.456	35.374	35.374
Rat						100	33.333	36.054	34.014
Opossum							100	40.136	39.456
Duck								100	94.5578
Gallus									100

**Table 11:** The correlation and significance analysis between our similarity analysis results of beta globin protein sequences in Table 8 and PID% similarity matrix in Table 10.

	Correlation coeff. (*r*) of our approach	*t*-value of our approach
Human	−0.8974	5.3806
Gorilla	−0.8715	4.7015
Chimp	−0.9105	5.8266
Lemur	−0.9151	6.0024
Mouse	−0.8489	4.2505
Rat	−0.7248	2.7830
Opossum	−0.5318	—
Duck	−0.7169	2.7209
Gallus	−0.6960	—

**Table 12:** The similarity distance for nine different species of ND5 proteins calculated by the ClustalW.

	Human	Gorilla	P. chimp	C. chimp	F. whale	B. whale	Rat	Mouse	Opossum
Human	0	10.7	7.1	6.9	41	41.3	50.2	48.9	50.4
Gorilla		0	9.7	9.9	42.7	42.4	51.4	49.9	54
P. chimp			0	5.1	40.1	40.1	50.2	48.9	50.1
C. chimp				0	40.4	40.4	50.8	49.6	51.4
F. whale					0	3.5	45.3	46.8	52.7
B. whale						0	45	45.9	52.7
Rat							0	25.9	54
Mouse								0	50.8
Opossum									0

**Table 13:** The correlation and significance analysis between our similarity analysis results of ND5 protein sequences in Tables 9 and 7 in [33] and Table 3 in [15] and ClustalW similarity matrix in Table 12.

	Correlation coeff. (*r*) of our approach	*t*-value of our approach	Correlation coeff. (*r*) of [33]	*t*-value of [33]	Correlation coeff. (*r*) of [15] (Table 3)	*t*-value of [15] (Table 3)
Human	0.9159	6.0389	0.7819	3.3181	0.9419	7.4169
Gorilla	0.9062	5.6692	0.7630	3.1229	0.9363	7.0524
P. chimp	0.8811	4.9288	0.7856	3.3588	0.8755	4.7944
C. chimp	0.9345	6.9482	0.7808	3.3069	0.9448	7.6311
F. whale	0.9674	10.109	0.8360	4.0314	0.8146	3.7160
B. whale	0.9239	6.3875	0.8430	4.1463	0.6593	—
Rat	0.8048	3.5871	0.9213	6.2663	0.6479	—
Mouse	0.8112	3.6699	0.6391	—	0.6308	—
Opossum	0.6378	—	0.4299	—	0.4772	—

## Data Availability

All data are mentioned clearly in the manuscript in Section 2 under the title “Dataset, Technology, and Tools.” In this section, we illustrate the data in three tables: Tables 1, 2, and 3. We also mention that data are downloaded from “Gene Bank.” All data files are with extension“, fasta”.
